# Surface Oxidation of Cu_2_O Nanoparticles by Adsorbed Ammonia

**DOI:** 10.3390/nano12234242

**Published:** 2022-11-29

**Authors:** Siwoo Lee, Ji Won Jang, Young Bok Ryu

**Affiliations:** 1 Korea Institute of Industrial Technology (KITECH) Ulsan Division, Ulsan 44413, Republic of Korea; 2 Hyundai Motors, Ulsan 44259, Republic of Korea

**Keywords:** Cu_2_O nanoparticle, surface analysis, oxidation, antibacterial activity, ammonia, azide

## Abstract

Copper-based nanoparticles have been intensively studied owing to their superior antibacterial activity. In this study, cuprous oxide (Cu_2_O) nanoparticles were synthesized using two different methods. In particular, two methods for synthesizing copper oxide from NaOH, namely, with and without the addition of NH_3_, were used to adjust the morphology of the nanoparticles. The nanoparticles from the NH_3_ and NaOH samples possessed an octahedral morphology. The crystal structure of the samples was confirmed by X-ray diffraction. The size distribution of the NH_3_ sample was narrower than that of the NaOH sample. Furthermore, the average size of the NH_3_ sample was smaller than that of the NaOH sample. Unexpectedly, the antibacterial activity of the NH_3_ sample was found to be lower than that of the NaOH sample. X-ray photoelectron spectroscopy and Fourier-transform infrared spectroscopy revealed that the adsorbed NH_3_ caused the surface oxidation of Cu_2_O nanoparticles with azide (N_3_) formation on surface.

## 1. Introduction

Because of the recent pandemic, wearing a mask has become a daily routine. Consequently, there have been periods when mask prices have become extremely high owing to the sold-out phenomenon, resulting in an increase in the reuse rate of masks [1]. However, although masks play a role in filtering viruses or bacteria, they do not kill bacteria or viruses. In addition, reusing masks is undesirable because the humid and warm internal conditions due to exhalation create an environment in which bacteria can thrive [2]. To prevent the growth of bacteria due to such contamination, research has been conducted to impart antibacterial activity to filter materials. Such studies have mainly involved the use of silver nanoparticles to impart antibacterial properties. Therefore, the United States Food and Drug Administration has recently begun to regulate the use of silver nanoparticles [3]. Meanwhile, the United States Environmental Protection Agency confirmed that copper and its alloys were the first metallic antimicrobial agent [4]. Thus, research on antibacterial agents that could replace silver has become active. Copper has been used for antibacterial purposes since ancient times owing to its excellent antibacterial activities. It is cheaper than silver and exists abundantly on earth; therefore, many studies on the development of antibacterial agents using copper have been conducted [5,6,7,8,9,10,11,12,13,14,15,16,17]. In particular, inorganic antibacterial agents are usually produced and used with nanometer size to increase the surface area. Nanoparticles are mainly synthesized using methods such as the chemical reduction method [18,19,20,21,22] or hydrothermal synthesis method [23,24]. Studies have been conducted to control the size and shape of particles using the chemical reduction method because the properties of nanoparticles depend on their size, shape, crystal structure, etc. [25,26,27,28]. Copper undergoes oxidation when exposed to the atmosphere [29,30]. Thus, if produced in nanoparticle form, its contact area with oxygen increases, leading to faster oxidation; this makes maintaining the antibacterial properties of copper difficult. Oxidized copper is classified into two types: cuprous oxide (Cu_2_O) and cupric oxide (CuO). Cu_2_O is superior to CuO in terms of antibacterial activity [31]. In particular, the catalytic activity, antibacterial activity, and adsorption characteristics of Cu_2_O nanoparticles have been reported to be excellent when the crystal plane (111) is present on the surface of the particles [32,33,34]. Although mechanisms of the antibacterial activity of copper have not been clearly identified, it is generally known to be caused by direct contact between copper and bacterial cells [4,9]. Although antibacterial and adsorption properties depend on the surface properties of nanoparticles, most studies measured only the crystal information of nanoparticles through X-ray diffraction (XRD) and did not analyze the surface properties. This may cause the antibacterial activity of the synthesized Cu_2_O to be incorrectly analyzed. When NH_3_ was included among materials added to control the shape of Cu_2_O particles, the antibacterial performance was diminished compared to the particles synthesized using other methods. The present study confirmed the reason for the decreased antibacterial properties of Cu_2_O octahedral nanoparticles prepared using two different methods.

## 2. Materials and Methods

The synthesis of Cu_2_O octahedra was performed using two methods. The first method used NH_3_ as an additive. A difference in growth rate in the <100> direction compared to the <111> direction was generated by controlling the molar ratio of NH_3_ and copper ions; accordingly, spherical and octahedral particles could be synthesized [32]. The <100> direction was parallel to the x-, y-, and z-axis, and the <111> directions were perpendicular to the (111) facet groups. In the other method, by increasing the amount of NaOH to be added and adsorbing an extra OH^−^ group on the (111) crystal plane, a Cu_2_O octahedron with dominant crystal growth in the <100> direction could be synthesized [35].

Cu(NO_3_)_2_∙3H_2_O (99–104%) and hydrazine hydrate (N_2_H_4_, 50–60%) reagents were purchased from Sigma-Aldrich (Merck Co., Ltd., Darmstadt, Germany). Furthermore, NaOH beads (97%) and ammonium hydroxide (25–28%) were purchased from DAEJUNG (Seoul, Korea). All materials were used without additional purification processes.

The entire Cu_2_O nanoparticle synthesis was performed while maintaining a temperature of 15 °C using a beaker with a double jacket and a chiller, and stirring at 300 rpm using an overhead stirrer. In the octahedral nanoparticles prepared by adding NH_3_ (NH_3_ sample), 4.5 mL of 14.03 M NH_3_ was added to 180 mL of a 0.05 M solution of Cu(NO_3_)_2_·3H_2_O and stirred, and 18 mL of 1 M NaOH was added dropwise. The solution was stirred for 15 min, and 1.5 mL of 17.66 M N_2_H_4_, a reducing agent, was added and stirred for 90 min. In the synthesis using only NaOH (NaOH sample), 42.2 mL of 1 M NaOH was added dropwise to 360 mL of a 0.025 M Cu(NO_3_)_2_·3H_2_O solution and stirred for 15 min. Subsequently, 1.875 mL of 17.66 M N_2_H_4_ was added and stirred for 2 h. The synthesized nanoparticles were washed 3–4 times with deionized (DI) water and ethanol and vacuum-dried.

The synthesized sample was analyzed using a scanning electron microscope (SEM, Hitachi SU820, Hitachi. Co., Ltd., Tokyo, Japan) to confirm particle size and shape, and XRD (/MAX 2500-V/PC, Rigaku Co., Ltd., Tokyo, Japan) was employed to acquire a diffraction pattern using a Cu-Kα wavelength to obtain crystal parameter values through peak analysis. Fourier-transform infrared spectroscopy (FT-IR Varian, 670, Varian Inc., CA, USA) was performed to analyze the vibration pattern of the material adsorbed on the surface. For the surface analysis of the nanoparticles, X-ray photoelectron spectroscopy (XPS, Thermo Scientific Nexsa, Thermo Fisher Scientific, Middlesex County, MA, USA) measurements were performed, and the core level shift was analyzed to obtain the information of oxidation states between Cu and O atoms. The X-ray source type used was the Microfocus monochromatic Al-Kɑ (1486.6 eV) X-ray source.

To compare the antibacterial properties of the synthesized nanoparticles, the colony-forming units (CFU) of general bacteria were counted and evaluated using the dry medium film method. The antimicrobial evaluation method was as follows: first, bacteria present on the hand were collected using a pipette swab and diluted 10 from 1 to 10^4^ times using a sterile dilution solution; then, 1 mL of the diluted solution was inoculated in each dry medium and cultured in an incubator at 37 °C for 24 h. One colony obtained from the previous culture was placed in 9 mL of buffered peptone water (BPW) solution using a sterilized loop and subjected to primary incubation for 24 h under the same conditions as mentioned above. One milliliter of the cultured solution was placed in 9 mL of the new BPW solution and incubated for a second time for 24 h, and the second cultured solution was diluted 10 times to the power of 1–3 and incubated in a dry medium to obtain the standard bacterial solution. In the experimental group, 0.01 g of the synthesized nanoparticles was dispersed in 30 mL of DI water, mixed with a standard bacterial solution at a volume ratio of 1:1, and cultured. In the control group, DI water was added to the standard bacterial solution in the same way as in the experimental group and cultured to compare the number of CFUs.

## 3. Results and Discussion

To confirm the crystal structure and component, XRD measurement was conducted on the Cu_2_O nanoparticles synthesized using the two methods. The crystal structure was confirmed using the International Center for Diffraction Data (ICDD) library.

**Figure 1:**
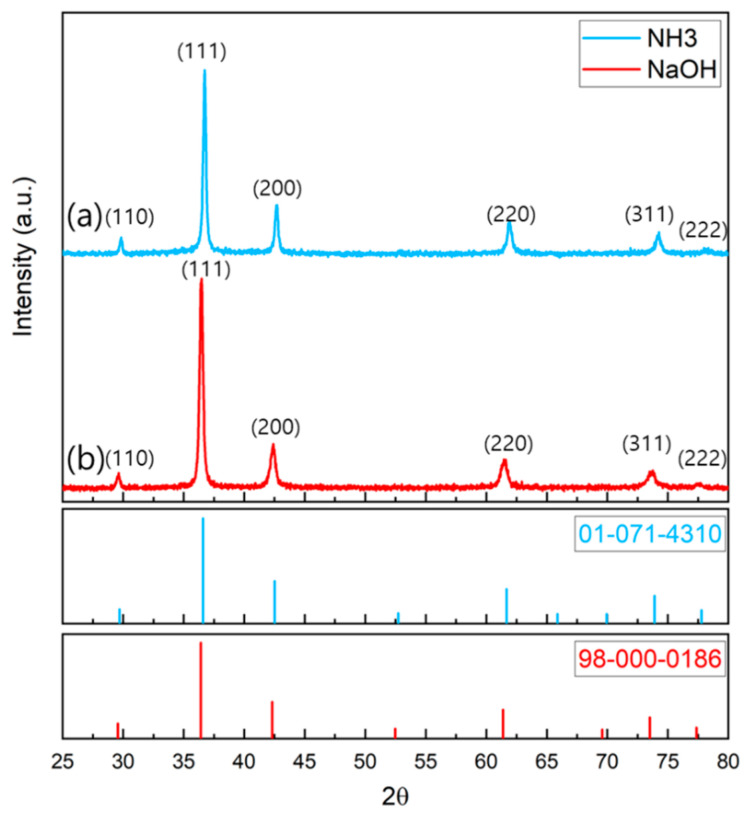
XRD patterns of Cu_2_O NPs (**a**) with NH_3_ (ICDD PDF#01-071-4310) and (**b**) without NH_3_ (ICDD PDF#98-000-0186).

**Figure 2:**
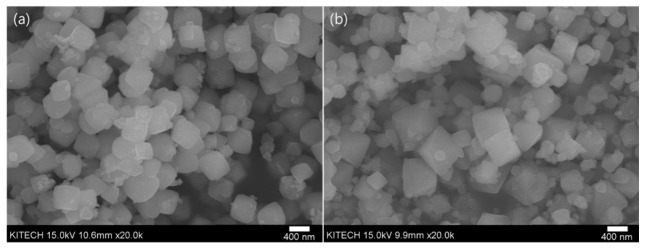
SEM images of Cu_2_O NPs (**a**) with NH_3_ and (**b**) without NH_3_.

As shown in Figure 1, the XRD pattern of the particles synthesized by adding NH_3_ (hereinafter, NH_3_ sample) matched the crystal pattern of ICDD PDF <01-071-4310> Cu_2_O, and the XRD pattern of the particles synthesized by adding NaOH (hereinafter, NaOH sample) matched the crystal pattern of ICDD PDF <98-000-0186> Cu_2_O; thus, the crystal structures of both samples were confirmed. The average crystal size was obtained from the Scherrer equation using the full width at half maximum obtained through the analysis of the crystal planes of the main peaks (111) and (200) of the XRD patterns, and the d-spacing value was obtained using Bragg’s law. The crystal sizes of the NH_3_ and NaOH samples were 19 and 25.04 nm, respectively; thus, the crystal size was larger when only NaOH was used. The size distribution of the nanoparticles verified from the SEM images shown in Figure 2 was 300–450 and 250–730 nm for NH_3_ and NaOH, respectively; the size distribution of the NaOH sample was wider than that of the NH_3_ sample.

**Figure 3:**
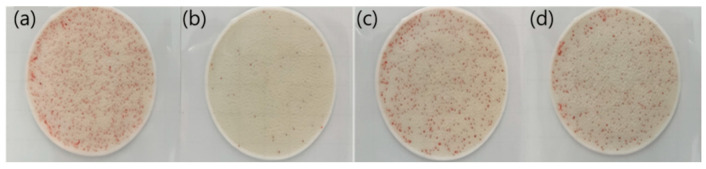
Antibacterial activity of (**a**) control, (**b**) NaOH sample (**c**) NH_3_ sample, and (**d**) 80 °C treated NaOH sample.

The antibacterial evaluation presented in Figure 3 and Table 1 reveals that an average of 706.8 colonies were formed in the control group (a) and 54.3 colonies were formed in the NaOH sample (b). By contrast, the number of colonies in the NH_3_ sample (c) significantly increased to 466.3 compared to that in the NaOH sample. This is an unexpected result because the NaOH sample had a larger average size of nanoparticles compared with the NH_3_ sample; thus, its antibacterial activity was expected to be worse than that of the NH_3_ sample. When the NaOH sample was thermally treated at 80 °C, 465.5 colonies (a higher value than in the untreated sample) were obtained. The surface oxidation of the Cu_2_O particles was assumed to be the reason for the decreased antibacterial activity of the NH_3_ sample.

**Figure 4:**
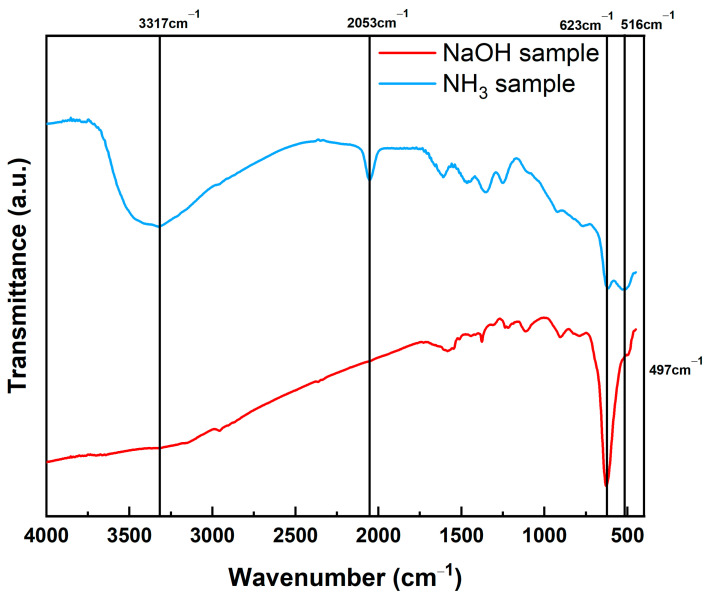
FT-IR spectra of the NH_3_ and NaOH samples.

Figure 4 shows the FT-IR spectra of the NH_3_ and NaOH samples. In the spectrum of the NaOH sample, Cu_2_O and CuO, represented by 623 cm^−1^ [36] and 497 cm^−1^ [37], respectively, were observed. However, in addition to the Cu_2_O peak at 623 cm^−1^ and the CuO peak at 516 cm^−1^ [38] in the spectrum of the NH_3_ sample, a new peak appeared at 2053 cm^−1^, which was close to 2030 cm^−1^, the peak that was observed in Busca et al.’s study [39]. This peak is attributed to the azide (N_3_) species. In addition, a N–H stretching peak at 3317 cm^−1^ was observed [40].

**Figure 5:**
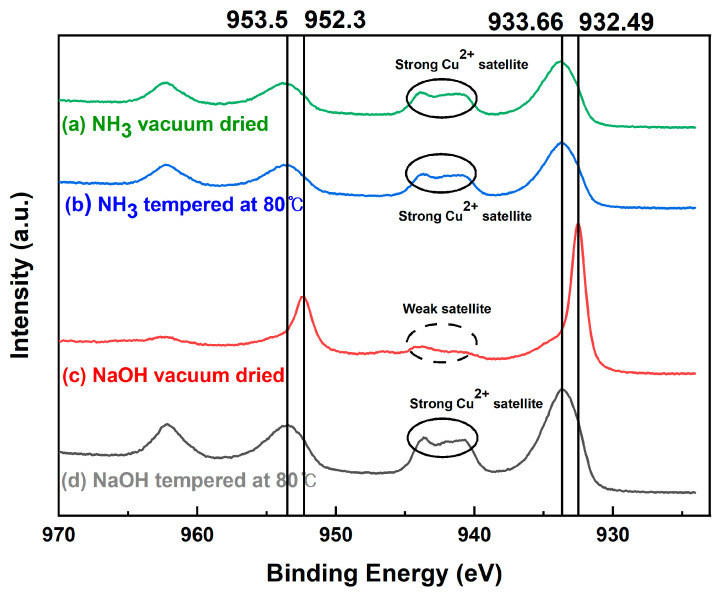
XPS spectra of Cu_2_O NPs: (**a**) NH_3_ sample dried in vacuum, (**b**) NH_3_ sample tempered at 80 °C, (**c**) NaOH sample dried in vacuum, and (**d**) NaOH sample tempered at 80 °C.

To investigate the surface properties of the nanoparticles, XPS measurement, which could be used to analyze the surface information to a depth of 10 nm, was applied to the samples. Figure 5 shows the binding energy of the Cu 2p_3/2_ region according to the presence or absence of 80 °C heat treatment of the NH_3_ and NaOH samples. The vacuum-dried NH_3_ and NaOH samples appeared as Cu_2_O in the XRD pattern shown in Figure 1. However, surface analysis with XPS revealed that the NH_3_ sample had a strong Cu^2+^ satellite peak between 940 and 945 eV and peaks at 933.66 and 953.5 eV; thus, CuO could be inferred to exist on the surface. By contrast, the NaOH sample had a Cu_2_O peak at 932.48 eV and the bonding of Cu_2_O existed at 952.3 eV, indicating that the surface was Cu_2_O [41].

**Figure 6:**
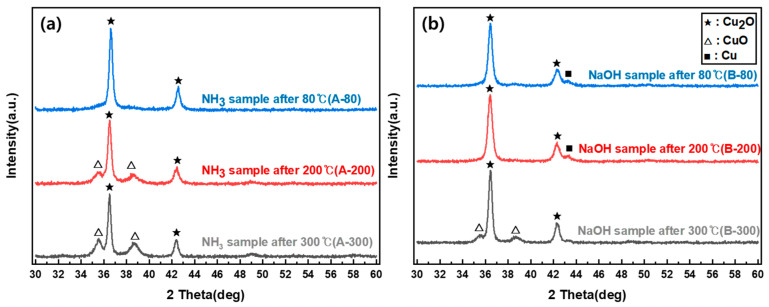
XRD patterns after heat treatment: (**a**) NH_3_ samples; (**b**) NaOH samples.

In the case of heat-treated samples at 80 °C, the surface of both samples was oxidized, forming a CuO layer. In the crystal structure obtained from XRD measurement after thermal treatment with different temperatures to compare the oxidation stability (Figure 6), the NH_3_ sample showed a CuO crystal phase from 200 °C, while the NaOH sample showed a CuO crystal phase after 300 °C; the thermal oxidation stability of the NaOH sample was better than that of the NH_3_ sample. This is consistent with the preceding results from XPS, in which the surface of the NH_3_ sample was already oxidized to form CuO.

**Figure 7:**
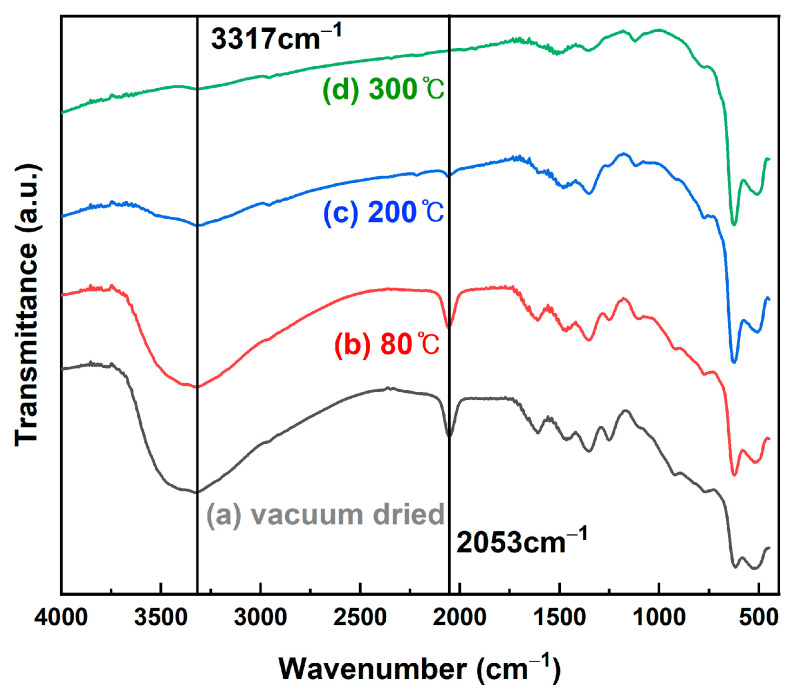
FT-IR spectra of NH_3_ samples under (**a**) vacuum drying and heat treatment at (**b**) 80 °C, (**c**) 200 °C, and (**d**) 300 °C.

As illustrated in Figure 7, the peaks related to the azide (N_3_) group and a wide N–H group of 3317 cm^−1^ formed in the NH_3_ sample decreased gradually as the heat treatment temperature increased, and all peaks disappeared after the heat treatment at 300 °C. The phenomenon in which an azide group is formed on the Cu_2_O surface is assumed to occur when NH_3_ is decomposed while being adsorbed on the Cu_2_O surface. A previous study reported that the formation of an azide group after N_2_H_4_ adsorbed on the TiO_2_ surface was decomposed on the surface with NH_3_ [42]. 

The CuO shell formation of Cu_2_O nanocrystals is considered to be similarly associated with the adsorption of NH_3_. Incidentally, a study reported that NO_x_ is reduced using NH_3_ and Cu_2_O catalysts [43]. Because Cu(NO_3_)_2_ is used as the precursor, sufficient NO_3_^−^ ions are present around it. Therefore, NH_3_ and NO_3_^−^ ions present on the metal surface are assumed to interact and reduce to NO_2_; accordingly, Cu_2_O is oxidized to form CuO on the surface. The result that NO_3_^−^ ions adsorbed on the metal surface are reduced to NO_2_^−^ ions has been reported [44].

When the NH_3_ sample was heated at heat treatment temperatures of 80 °C, 200 °C, and 300 °C, the peaks of the azide group of 2053 cm^−1^ formed on the surface gradually decreased in size as the temperature increased and disappeared at 300 °C. In addition, the N–H plane observed at 3317 cm^−1^ disappeared equally.

## 4. Conclusions

This study revealed that, when NH_3_ is added in the synthesis of Cu_2_O nanoparticles, a CuO shell is formed on the surface of the Cu_2_O nanoparticles. Consequently, the antibacterial activity of the nanoparticles from the NH_3_ sample was lower than that of the Cu_2_O nanoparticles prepared using only NaOH. When fabricating nanoparticles, which have important surface properties, NH_3_ could form unwanted oxides on the surface owing to redox reactions; thus, because XRD crystal analysis cannot reveal oxides formed on the surface, surface analysis techniques such as XPS must be performed simultaneously to accurately study nanoparticle properties. For further understanding oxidation caused by NH_3_ adsorption, using another copper precursor such as copper acetate (Cu(Ac)_2_) or copper chloride (CuCl_2_), or another reducing agent with NH_3_ could be part of future experiments. These are currently being considered for further investigation. In addition, to investigate the effect of CuO shell on various species such as Gram-positive (pyogenes, Strep. agalactiae, enterococci) or Gram-negative (Acinetobacter, E. coli, Klebsiella) bacteria, the antibacterial activity of each species could be further examined. Although the formed CuO shell on Cu_2_O nanoparticles diminished the antibacterial activity, the Cu_2_O/CuO core–shell nanoparticles are expected to be used as photoelectrochemical catalysts owing to the charge separation for organic degradation [45,46].

## Figures and Tables

**Table 1:** Average colony number of samples.

Sample	Control	NaOH	NH_3_	NaOH 80 °C
Colony count (average)	706.8	54.3	466.3	465.5

## Data Availability

Not applicable.
